# Development of the Leisure Activity Scale for young adults: Reliability and validity

**DOI:** 10.1002/pcn5.70070

**Published:** 2025-03-03

**Authors:** Rumiko Kinkawa, Miki Ono, Satoshi Horiuchi, Masayuki Kikkawa, Shunichiro Ito, Akifumi Shimasaki, Yu Tamada, Jiro Masuya, Mina Honyashiki, Takeshi Inoue

**Affiliations:** ^1^ Department of Psychiatry Tokyo Medical University Tokyo Japan; ^2^ Department of Psychiatry Nishi‐Hachioji Hospital Tokyo Japan; ^3^ Department of Social and Clinical Psychology Hijiyama University Hiroshima Japan; ^4^ Department of Psychiatry Tokyo Medical University Hachioji Medical Center Tokyo Japan

**Keywords:** anxiety, leisure activity, recovery experience, resilience, well‐being

## Abstract

**Aim:**

Previous studies have documented the beneficial effects of leisure activities on mental health. However, limited methods or scales are available to measure leisure activities for the evaluation of mental health or the intervention of mental disorders, and data on the therapeutic benefits of leisure activities for mental health are scarce. Furthermore, few studies have investigated the effects of leisure activities on the mental health of young individuals. Therefore, we developed the Leisure Activity Scale (LAS; a 7‐item, 5‐point scale) to assess the leisure activities of young adults, and aimed to confirm its reliability and validity.

**Methods:**

This study involved 551 Japanese participants aged 18–25 years and was conducted through an online survey from September to November 2022. Reliability of the LAS was assessed through internal consistency analysis. To determine convergent and discriminant validity, the LAS was compared with various scales measuring psychometric properties, including depression, anxiety, work fatigue recovery, resilience, neuroticism, emotion regulation, well‐being, and ill‐being.

**Results:**

Factor analysis indicated that the 7‐item LAS was the most appropriate measure for use in mental health (Cronbach's α coefficient = 0.756). Total LAS7 scores positively correlated with recovery experience, resilience, emotion regulation, and well‐being, and negatively correlated with state anxiety. These were all weak correlations, with correlation coefficients of less than 0.3. Total LAS7 scores did not correlate with trait anxiety, neuroticism, or ill‐being.

**Conclusions:**

Our results demonstrated the reliability of the LAS, but its convergent validity was insufficient. The LAS can comprehensively assess leisure activities, although further improvement regarding its validity is needed.

## Graphical abstract

Previous studies have highlighted the mental health benefits of leisure activities but lack specific measurement tools for psychiatric practice, particularly for younger individuals. To address this, we developed the Leisure Activity Scale (LAS), a 7‐item, 5‐point scale, and validated it among 551 young Japanese volunteers. The LAS demonstrated strong internal consistency (Cronbach's alpha: 0.756) and significant correlations with positive mental health outcomes, confirming its efficacy as a tool for assessing leisure activities in young adults.

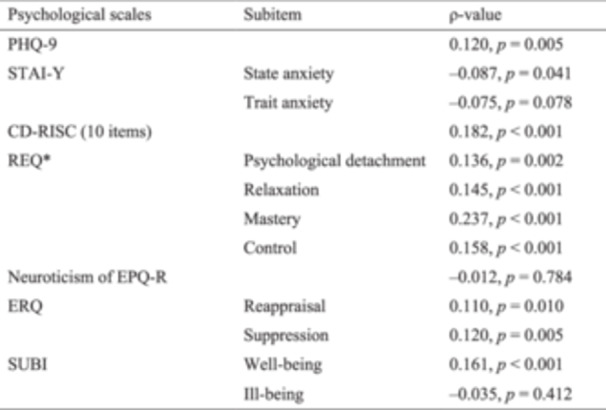

## INTRODUCTION

Leisure activities are activities that individuals engage in for pleasure and well‐being, which are detached from work and daily routines.^1^ Prior research has demonstrated that leisure activities positively influence mental health. Leisure activities are negatively correlated with depressive symptoms,^2^ and positively correlated with subjective well‐being.^3^ Leisure coping has been reported to reduce depressive symptoms.^4^ A large number of leisure activities have been shown to improve depressive symptoms via resilience,^5^ and to reduce stress.^6^ In daily psychiatric practice, information regarding the extent to which patients engage in leisure activities may be helpful for disease diagnosis, occupational therapy, or behavioral activation therapy as part of cognitive behavioral therapy.^7^ In contrast, therapeutic or diagnostic lines of evidence regarding the effects of leisure activities on mental health are scarce. Thus, to clarify the effects of leisure activities on general mental health, mental symptoms, and mental disorders, quantitative analysis of the effects of leisure activities on mental symptoms is required, to obtain scientific lines of evidence.

From the perspective of mental health care, various issues have been recognized in prior studies regarding the methods of evaluating leisure activities or determining their effectiveness. First, most methods of assessing leisure activities in previous studies have quantified the level of physical activity performed during leisure time; that is, these studies have measured the frequency, quantity, and intensity of physical activities during the leisure time, rather than analyzing the content of the leisure activity itself.^8^, ^9^, ^10^ In contrast, the Victorian Longitudinal Study is a comprehensive assessment tool with 57 items and 11 activity factors that cover various leisure activities other than physical activities; however, the large number of assessment items may make applying this tool in mental health practice difficult.^11^ Conversely, the Pittsburgh Enjoyable Activities Test is a convenient assessment tool of leisure activity levels based on the total score; however, its content is biased and does not cover many types of leisure activities.^12^ Furthermore, most previous studies have focused on a wide range of age groups, including middle‐aged adults or elderly adults, and have not adequately considered the leisure activities performed by young adults.^1^, ^11^, ^12^, ^13^ In summary, the previous methods for evaluating leisure activities had the following limitations: they did not cover a variety of leisure activities other than physical activities, they were not quantifiable and lacked simplicity, and they were difficult to apply to young adults.

Next, issues regarding the evaluation of the effects of leisure activities in previous research are as follows. First, assessment scales, such as the Center for Epidemiologic Studies Depression Scale (CES‐D) and the Hospital Anxiety and Depression Scale (HADS), were used to evaluate the effects of leisure activities on mental symptoms; however, these scales are not considered standard in psychiatry and have been insufficient for adequate assessments.^9^, ^11^, ^12^, ^13^ Second, the participants of previous studies on leisure activities were often elderly adults, so many of the studies have focused on their cognitive function and well‐being as outcomes rather than on mental symptoms.^3^, ^13^, ^14^ In light of the above, previous leisure activity rating scales have not been adequately validated for their application for the assessment of mental symptoms and mental health. To quantitatively assess the leisure activities of people with mental health care issues, validation of their association with various mental symptoms and personality traits is warranted.

In recent years, young adults, particularly those between 18 and 29 years old, have gained attention as they are in the emerging adulthood stage, which is the most mentally unstable period of life.^15^, ^16^ Emerging adults have a high incidence of anxiety and mood disorders,^17^ and depression in particular leads to the serious problem of suicide risk.^18^ As previous studies have reported that the use of antidepressants in emerging adults increases suicidal adverse events, considering the risk of suicide, medication is strongly discouraged.^19^ Hence, the first‐priority approach may be an intervention that works with the lifestyles of emerging adults (e.g., preventive mental health measures or behavioral activation therapy).^20^ At present, however, there are few studies on the effects of leisure activities in emerging adults, and no valid quantitative assessment methods applicable to them have been proposed.

Considering the above background and the paucity of evidence, the purpose of this study was to develop a quantitative leisure activity scale that comprehensively assesses a variety of leisure activities applicable to young adults, and to test its validity and reliability as well as its association with psychological characteristics. To make the scale specific and simple, the content of the activity items was classified into 10 categories, including a wide range of leisure activities. Leisure activities not covered by the scale for elderly adults, such as social networking services (SNSs), videos, and online games, were also included in the survey. The factor structure of the 10‐item Leisure Activity Scale (LAS) was investigated by exploratory factor analysis. As a method of quantification, we used a 5‐point scale, in which the participants reported the frequency by which they performed each activity, and the total score was considered to represent the overall amount of leisure time activities. We investigated the correlation between the participants' total scores on our LAS and various psychological measures, including depression, well‐being, anxiety, resilience, recovery experiences associated with coping with work, and emotion regulation.

## METHODS

### Participants

Participants were recruited from September to November 2022 through an online marketing research company (Macromill, Inc.). Drawing from the company's database of 1.07 million individuals aged 18–25 years, we selected those who were unmarried, childless, and native Japanese speakers. We then conducted a random sampling stratified by region, gender, and age. This process resulted in a sample representative of the target demographic, and we subsequently emailed this group. Those who accessed the link were provided with a detailed study overview, including objectives, methodology, potential benefits, and risks. By beginning to answer the survey, participants were considered to have given their consent. Individuals participating in the present study were paid volunteers. A total of 551 participants (18–25 years old, 246 men, 301 women, sex unreported for 4) were included in the analysis. These participants were considered to be at the age of emerging adulthood.^15^ In this study, the data provided by an Internet research company were pseudonymized and non‐personally identifiable, and did not contain any personal information about the participants. This study was conducted in accordance with the Declaration of Helsinki and approved by the Medical Ethics Review Committee of Tokyo Medical University (study approval number: T2022‐0053).

### Questionnaires

Data were collected via the Internet from participants who completed self‐administered forms for demographic information, the Leisure Activity Scale, and the psychometric questionnaire.

#### Leisure Activity Scale

As a clarification of the concept of leisure activities, this study used Verghese's definition of “leisure,” which defines leisure time activities as activities that individuals engage in for enjoyment or well‐being, which are independent of work or activities of daily living.^1^ In developing the LAS, several emerging adults were consulted regarding the content of their leisure activities, and the applicability of these activities to emerging adults was preliminarily investigated. SNSs, videos, and online games were included as leisure activities of young adults, from the Leisure White Paper 2021 of Japan.^21^ Finally, as a pilot study, an interview survey was conducted on 10 university students to check whether there were any shortcomings in the youth activities included, to revise or add items, and to check the communicability of the questions. Based on the study by Moriyama and Doi,^22^ leisure activities were classified into 10 categories (Meeting and interacting type, Spiritual type, Tour type, Thinking/Learning type, Physical activities type, Cultural activities–Creative type, Cultural activities–Appreciation type, Social activities type, Feeling nature type, and Information‐gathering type) (Table S1). Each category includes three to 15 activities, with a total of 68 activities, as presented in Table S1. The frequency of each activity was rated on a 5‐point scale (*only occasionally* or *not at all* = 1 point, *1–3 times a month* = 2 points, *once a week* = 3 points, *several days a week* = 4 points, and *almost every day* = 5 points). Leisure activity total scores were calculated by summing the scores of the seven categories that were determined by factor analysis (shown in the Results section), and were considered to represent the overall amount of leisure time activities (ranging from 7 to 35 points). The English version and Japanese version of the LAS are shown in Tables S1 and S2, respectively.

#### Patient Health Questionnaire‐9

Depressive symptom severity was assessed using the Japanese version of the Patient Health Questionnaire‐9 (PHQ‐9), which is also widely used as a screening tool for major depressive disorder.^23^, ^24^ Higher total scores of the PHQ‐9 indicate more severe depressive symptoms. The Cronbach's α coefficient for the total score of the PHQ‐9 was 0.95, indicating excellent internal consistency.

#### State–Trait Anxiety Inventory Form Y

The Japanese version of the State–Trait Anxiety Inventory Form Y (STAI‐Y) was used to assess the severity of state and trait anxiety.^25^, ^26^ The two subscales of the STAI‐Y are State Anxiety (anxiety that occurs temporarily in response to an event; one's current state) and Trait Anxiety (personality tendency to be anxious; one's usual state). Higher total scores of each subscale indicate higher anxiety. The Cronbach's α coefficients for the total scores of the State Anxiety subscale and Trait Anxiety subscale were 0.90 and 0.89, respectively, indicating good internal consistency.

#### Connor–Davidson Resilience Scale

The Japanese version of the Connor–Davidson Resilience Scale (CD‐RISC) was used to assess resilience.^27^, ^28^ In the present study, the CD‐RISC‐10 was used,^29^ which consists of 10 items extracted from the 25 items of the CD‐RISC, namely, questions 1, 4, 6, 7, 8, 11, 14, 16, 17, and 19. The total CD‐RISC‐10 score was used for the analysis in the present study. The Cronbach's α coefficient for the total CD‐RISC‐10 score was 0.95, indicating excellent internal consistency.

#### Recovery Experience Questionnaire

To measure the index of “recovery experiences,” we used the Japanese version of the Recovery Experience Questionnaire (REQ).^30^, ^31^ The REQ assesses how individuals recover from work‐associated fatigue during their leisure time after a workday. The questionnaire consists of four subscales—Psychological Detachment, Relaxation, Mastery, and Control—each with four items. Higher scores on these subscales indicate effective recovery from fatigue. In this study, responses were collected from employed respondents regarding after‐work activities, and from students regarding after‐class activities. Unemployed individuals were excluded from the analysis. The Cronbach's α coefficients for the total scores of the Psychological Detachment, Relaxation, Mastery, and Control subscales were 0.85, 0.91, 0.89, and 0.90, respectively, indicating good internal consistency.

#### Neuroticism subscale of the Eysenck Personality Questionnaire‐Revised

To assess neuroticism, a typical personality trait, as an indicator of an individual's vulnerability to depression and other psychiatric disorders, the shortened version of the Neuroticism subscale of the Japanese Eysenck Personality Questionnaire‐Revised (EPQ‐R) was used.^32^, ^33^ The Cronbach's α coefficient for the Neuroticism subscale was 0.90, indicating good internal consistency.

#### Emotion Regulation Questionnaire

The Japanese version of the Emotion Regulation Questionnaire (ERQ) was used to assess emotion regulation.^34^, ^35^ The ERQ has the following two subitems: (1) Reappraisal (which refers to regulating the emotion itself by interpreting events and situations that may cause the emotion to be expressed), and (2) Suppression (which refers to regulating the emotion felt at the time so that it is not expressed). The total score for each subitem was used for analysis. The Cronbach's α coefficients for total scores of the Reappraisal and Suppression subscales were 0.91 and 0.81, respectively, indicating good internal consistency.

#### Subjective Well‐Being Inventory

The Japanese version of the Subjective Well‐Being Inventory (SUBI) developed by the World Health Organization was used as the assessment scale for well‐being.^36^, ^37^ The SUBI has two subitems, Well‐being and Ill‐being, with higher scores indicating better health. In this study, all participants were unmarried and without children; thus, the analysis excluded questions about marriage and children. The Cronbach's α coefficients for total scores of the Well‐being and Ill‐being subscales were both 0.92, indicating excellent internal consistency.

#### Demographic information

The participants were asked to provide their demographic attributes, including age, sex, student or employment status, past history of mental illness, currently treated mental illness, family history of mental illness, and currently treated physical illness. Regarding education and paid labor, responses were sought based on student classification or occupation (1: high school/technical college students, 2: junior college/vocational school students, 3: university students, 4: graduate students, 5: full‐time employees, 6: part‐time employees, and 7: others), which were then reclassified into the following three categories: (1) students, (2) employed, and (3) unemployed. The respondents were asked to answer the binary question with a *Yes* = 2 or *No* = 1 for past history of mental illness and currently treated mental illness, family history of mental illness, and currently treated physical illness. The respondents were also asked to respond to the Subjective Social Status (SSS) Questionnaire, which measures the subjective perception of one's position in the social hierarchy, between 10 (*highest*) and 1 (*lowest*).^38^

### Data analysis

Statistical analysis was performed using SPSS Statistics Version 28 software (IBM, Armonk, NY, USA), with a *p*‐value of less than 0.05 considered to indicate a statistically significant difference between groups. Means and standard deviations for age and SSS were calculated for demographic information of the 551 emerging adult volunteers. Frequencies were obtained for sex, student or employment category, past history of mental illness, currently treated mental illness, family history of mental illness, and currently treated physical illness. A principal axis exploratory factor analysis was conducted using the promax rotation method to evaluate the construct validity of the LAS. The criterion of factor loading was set at 0.4 or more. A scree test was performed to determine the number of required factors.^39^ The LAS total scores and the mean ± standard deviation of each subitem of the LAS were also calculated. In addition, the means and standard deviations of the variables on the various psychological scales were calculated. Associations or correlations between the LAS total scores and demographic characteristics were analyzed using the Mann–Whitney *U*‐test, Kruskal–Wallis test (multiple comparisons followed by Dunn's test), or Spearman's correlation analysis. The LAS total scores and various psychological scale scores were used to analyze their correlation between total scores and various psychological scale scores (PHQ‐9, STAI‐Y, CD‐RISC, REQ, EPQ‐R, ERQ, and SUBI) by Spearman's correlation analysis. The internal consistency (Cronbach's α coefficient) was evaluated to verify the reliability of the LAS.

## RESULTS

### Participants’ demographic information and scores for LAS and other psychometric scales

Table 1 shows the demographic information of the participants. The average age of the participants was 22.0 ± 2.1 years (18–25 years). Other details, such as employment status, past history of mental illness, currently treated mental illness, currently treated physical illness, family history of mental illness in first‐degree relatives, and SSS, are also shown in Table 1. Table 2 shows the data from the psychometric scales: the PHQ‐9, STAI‐Y, CD‐RISC, REQ, EPQ‐R Neuroticism subscale, ERQ, and SUBI. Table 3 shows the data for the 10 subitems of the LAS.

**Table 1:** Demographic data of 551 emerging adult volunteers.

Characteristic or measure	Number or mean ± SD
Age (years)	22.0 ± 2.1
Sex (men : women)	246 : 301
Student or employment status (student : employed : unemployed)	255 : 245 : 51
Past history of mental illness (yes : no)	74 : 477
Currently treated mental illness (yes : no)	57 : 494
Currently treated physical illness (yes : no)	56 : 495
Family history of mental illness in first‐degree relatives (yes : no)	55 : 496
Subjective Social Status (1 lowest, 10 highest)	4.7 ± 2.0

Abbreviation: SD, standard deviation.

**Table 2:** Means and standard deviations of variables of various psychological scales (*N* = 551).

Psychological scales	Subitems	Mean ± SD
PHQ‐9	—	6.6 ± 6.5
STAI‐Y	State Anxiety	47.0 ± 10.7
	Trait Anxiety	49.1 ± 10.5
CD‐RISC (10 items)	—	18.9 ± 9.6
REQ^a^	Psychological Detachment	12.9 ± 3.8
	Relaxation	14.6 ± 3.9
	Mastery	12.0 ± 4.0
	Control	15.0 ± 3.9
EPQ‐R	Neuroticism	6.1 ± 4.1
ERQ	Reappraisal	24.4 ± 6.9
	Suppression	15.8 ± 4.7
SUBI	Well‐being	37.5 ± 8.3
	Ill‐being	45.6 ± 9.0

Abbreviations: CD‐RISC, Connor–Davidson Resilience Scale; EPQ‐R, Eysenck Personality Questionnaire‐Revised; ERQ, Emotion Regulation Questionnaire; PHQ‐9, Patient Health Questionnaire‐9; REQ, Recovery Experience Questionnaire; STAI‐Y, State–Trait Anxiety Inventory Form Y; SUBI, Subjective Well‐Being Inventory.

^a^ Data of 500 participants, excluding those who were unemployed.

**Table 3:** Leisure Activities Scale category scores.

Leisure Activities Scale category^a^	Mean ± SD
Meeting and interacting type	1.8 ± 1.2
Spiritual type	1.3 ± 0.8
Tour type	1.9 ± 1.0
Thinking/learning type	1.7 ± 1.1
Physical activities type	2.0 ± 1.3
Cultural activities–Creative type	1.8 ± 1.2
Cultural activities–Appreciation type	2.3 ± 1.4
Social activities type	1.3 ± 0.7
Feeling nature type	1.4 ± 0.9
Information gathering type	3.3 ± 1.7

^a^ Refer to Supplementary Table 1 for details of each category.

### Factor analysis of LAS

The scree plot showed that a two‐factor structure was appropriate for the LAS (Figure S1). Based on the criterion, factor loading was set at 0.40 or more, and seven items loaded more highly on Factor 1, whereas the other two items loaded more highly on Factor 2. However, because Factor 2 includes only two items, and the correlation coefficient between the items was 0.422, which was less than 0.7, we decided to omit Factor 2.^39^ In addition, factor loading of one item (i.e., the Physical activities type) was lower than 0.40. Accordingly, three items (i.e., Physical activities type, Cultural activities–Appreciation type, and Information‐gathering type) were removed, and the remaining seven items were used for the second exploratory factor analysis (principal axis factor analysis). The results of the analysis are shown in Table 4. Factor loadings of the seven items were all 0.40 or greater. Therefore, we finally used a 1‐factor model including seven items (LAS7) to calculate the total score of the LAS. LAS7 includes the items of Meeting and interacting, Spiritual, Tour, Thinking/Learning, Cultural activities–Creative, Social activities, and Feeling nature categories. Many of these activities are self‐initiated, and LAS7 represents active participatory behavior‐type activities. Cronbach's α coefficient for the LAS7 score was 0.756.

**Table 4:** Results of factor analysis (principal axis factoring) of Leisure Activity Scale 7 (LAS7, 7 categories).

Category	Factor loading
Meeting and interacting type	**0.426**
Spiritual type	**0.606**
Tour type	**0.503**
Thinking/Learning type	**0.553**
Cultural activities–Creative type	**0.518**
Social activities type	**0.728**
Feeling nature type	**0.547**

*Note*: Loadings with values greater than 0.4 are shown in bold.

### Correlations and associations between LAS7 total score and demographic and psychometric data

Nonparametric tests were used to analyze the correlations and associations between LAS7 total scores and demographic data (Table 5). LAS7 total scores were negatively correlated with age (*ρ* = –0.146, *p* < 0.001). LAS7 total scores differed significantly among the participants in the three social category groups (student, employed, and unemployed; *p* = 0.009). Multiple comparisons demonstrated that the student group had significantly higher LAS7 total scores than the employed group; however, there was no significant difference in the total scores between the employed and unemployed groups, or between the student and unemployed groups. LAS7 total scores were not associated or correlated with other demographic data.

**Table 5:** Correlations and associations between LAS7 total score and demographic data.

Characteristic or measure	Correlation or association with LAS7 total score
Age (years)	ρ ＝ –0.146, *p* < 0.001
Sex (men : women)	Men (11.0 ± 4.6) : women (11.18 ± 3.9), *p* = 0.089^a^
Student or employment status (student : employed : unemployed)	Student (11.6 ± 4.4) : employed (10.7 ± 4.1) : unemployed (10.5 ± 4.6), *p* = 0.009^b^
	Student vs employed: *p* = 0.030
	Student vs unemployed: *p* = 0.054
	Employed vs unemployed: *p* = 1.000
Past history of mental illness (yes : no)	Yes (11.4 ± 4.1) : no (11.0 ± 4.4), *p* = 0.190^a^
Currently treated mental illness (yes : no)	Yes (11.9 ± 4.5) : no (11.0 ± 4.3), *p* = 0.075^a^
Currently treated physical illness (yes : no)	Yes (11.4 ± 4.0) : no (11.1 ± 4.4), *p* = 0.304^a^
Family history of mental illness in first‐degree relatives (yes : no)	Yes (11.4 ± 3.8) : no (11.1 ± 4.4), *p* = 0.185^a^
Subjective Social Status (1: lowest; 10 highest)	ρ = –0.037, *p* = 0.388

Abbreviation: LAS, Leisure Activity Scale.

^a^ Mann–Whitney *U*‐test.

^b^ Kruskal–Wallis test.

ρ = Spearman's correlation coefficient.

Table 6 shows the correlations between LAS7 total scores and various psychometric scale scores. LAS7 total scores showed significant positive correlations with the scores of PHQ‐9, CD‐RISC, Reappraisal and Suppression of the ERQ, all four subscales of the REQ, and Well‐being of the SUBI; and LAS7 total scores showed significant negative correlations with State Anxiety of the STAI‐Y, but no significant correlations with Trait Anxiety of the STAI‐Y, Neuroticism of the EPQ‐R or Ill‐being of the SUBI.

**Table 6:** Correlation or association between LAS7 total scores and various psychological scale scores.

Psychological scales	Subitem	*ρ*‐value
PHQ‐9	—	0.120, *p* = 0.005
STAI‐Y	State Anxiety	–0.087, *p* = 0.041
	Trait Anxiety	–0.075, *p* = 0.078
CD‐RISC (10 items)	—	0.182, *p* < 0.001
REQ^a^	Psychological Detachment	0.136, *p* = 0.002
	Relaxation	0.145, *p* < 0.001
	Mastery	0.237, *p* < 0.001
	Control	0.158, *p* < 0.001
EPQ‐R	Neuroticism	–0.012, *p* = 0.784
ERQ	Reappraisal	0.110, *p* = 0.010
	Suppression	0.120, *p* = 0.005
SUBI	Well‐being	0.161, *p* < 0.001
	Ill‐being	–0.035, *p* = 0.412

^a^ Data of 500 participants, excluding those who were unemployed.

*Note*: ρ = Spearman's correlation coefficient.

Abbreviations: CD‐RISC, Connor–Davidson Resilience Scale; EPQ‐R, Eysenck Personality Questionnaire‐Revised; ERQ, Emotion Regulation Questionnaire; LAS, Leisure Activity Scale; PHQ‐9, Patient Health Questionnaire‐9; REQ, Recovery Experience Questionnaire; STAI‐Y, State–Trait Anxiety Inventory Form Y; SUBI, Subjective Well‐Being Inventory.

## DISCUSSION

To the best of our knowledge, this is the first study to investigate the association between leisure activities and various psychological characteristics using our newly developed LAS for emerging adults. The reliability of the LAS7 was suggested, as its Cronbach's α coefficient was relatively high. However, LAS7 scores demonstrated significant correlations with other pertinent self‐reported scales but the correlation coefficients were low (<0.3), which suggests that the hypothesis of convergent validity of the LAS7 was not clearly verified. Nevertheless, LAS7 total scores showed significant positive correlations with the experience of recovery from work‐associated fatigue, resilience, well‐being, and emotional regulation, and significant negative correlations with state anxiety. As mentioned in the Introduction section, leisure scales in previous studies were targeted at older people, focused on physical activity, lacked simplicity and quantifiability, and had insufficient validity in mental health care practice. The LAS that we developed may overcome such shortcomings of the previous scales, and may have novelty and strength in its comprehensive and quantitative presentation of the associations between leisure activities and various psychological characteristics in relation to mental health in emerging adults, such as mental symptoms and personality traits. Further studies are needed to investigate the utility of the LAS in the clinical setting.

In the present study, LAS7 total scores were negatively correlated with state anxiety of the STAI‐Y. In other words, this finding suggests that a higher frequency of leisure activities may result in reduced anxiety, or that lower levels of state anxiety are associated with more participation in leisure activities. A previous cross‐sectional study of college students reported the association between higher levels of state anxiety and a deficiency of leisure activities, although no definition or method of quantification of leisure activities was provided.^40^ Their results are consistent with the results of our present study. Another study investigating anxiety levels in college students before and after short‐duration leisure activities highlighted that chatting with friends and walking both significantly reduced state anxiety.^41^ Their results are also partially consistent with the findings of our present study, as chatting corresponds to the “Meeting and interacting” category of the LAS7. These results suggest that increasing leisure activities may alleviate anxiety in emerging adults.

Psychological scales were significantly correlated with LAS7 total scores. This indicates the following: the positive correlation of CD‐RISC scores suggests that participants who undergo more leisure activities have more resilience, that is, successful stress‐coping ability.^28^ The positive correlations of ERQ Reappraisal and Suppression scores suggest that participants who perform more leisure activities use more reappraisal strategies to regulate the occurrence of the emotion itself, by reinterpreting the event causing the emotion, and perform more strategies to suppress the expression of the emotion, respectively.^34^ The positive correlations of the four REQ subscales suggest that participants who perform more leisure activities recover more effectively from work‐ or school‐associated fatigue during their leisure time after a workday or school day, using four different strategies, namely, psychological detachment, relaxation, mastery, and control.^30^ The positive correlation of the SUBI Well‐being scores suggests that participants who perform more leisure activities show more tendencies for general well‐being‐positive affect, expectation‐achievement congruence, confidence in coping, and transcendence.

A surprising aspect of this study is that LAS7 total score showed a weak yet positive association with PHQ‐9 score, namely, depressive symptoms. The correlation coefficient of 0.120 is very weak, and hence may not be of practical significance. However, previous reports have indicated that leisure activities correlate with reduced depressive symptoms.^4^, ^9^, ^11^, ^12^, ^13^ It is hence plausible that leisure activity is not associated with fewer depressive symptoms in the participants in the present study, as LAS7 total scores were not significantly correlated with neuroticism, which is closely associated with depressive symptoms.^33^ As previous studies included a wide range of age groups or older adults, this suggests that the association between leisure activities and depressive symptoms in emerging adults may differ from that in other age groups; therefore, further replication/follow‐up investigations are warranted to confirm these findings. Furthermore, owing to the cross‐sectional design of our study, in which clinically depressed patients were not specifically included, the possibility cannot be excluded that emerging adults feeling depressive moods may actually choose more leisure activities as a method of coping.

We acknowledge some limitations in further generalizing the findings of the present study. First, our research focuses on young individuals aged 18 to 25 years; therefore, a future study will need to be performed to confirm whether or not the LAS is applicable for those older than 26 years, as well as middle‐aged and older adults. Second, the present study was a cross‐sectional study. Therefore, prospective trials should be performed to analyze the causal associations and mechanisms through which leisure activities affect anxiety, well‐being, emotion regulation, and resilience. Test–retest studies should be conducted in the future to confirm the reproducibility of the scale. Third, as the present study was conducted in young adult volunteers, in the future, the LAS should also be validated in patients with mental disorders. Fourth, as this research was conducted during the coronavirus disease 2019 (COVID‐19) pandemic in Japan (from February 2020 to May 2023), there is the possibility that scores on some LAS subitems may have been suppressed to a lower level, owing to the reduced amount of leisure activities involving outings, such as domestic sightseeing and travel. A review and re‐examination of leisure activities of young adults after the COVID‐19 pandemic will provide further essential data and lines of evidence, enabling the comparison of changes during and after the pandemic. Fifth, the unexpectedly weak positive correlation between LAS7 scores and depressive symptoms questions its practical significance. The weakness of the correlation suggests potential confounding factors. Sixth, the relatively small number of seven categories of the LAS7 may not adequately capture the diversity of leisure activities, although each category includes three to 15 activities, with a total of 50 activities. The LAS also does not consider cultural variations and age‐specific leisure pursuits. Seventh, a robust theoretical framework to contextualize the association between leisure activities and mental health outcomes was not established in this study. Therefore, further studies are needed to establish this theoretical framework. Finally, as the validity and reliability of the LAS were not sufficient, the LAS is at present a premature tool for use in clinical practice. There is much to be improved and verified before the LAS can be used in clinical practice.

## CONCLUSIONS

In this study, we developed the LAS, which is a brief self‐report leisure activity scale that comprehensively assesses leisure activities among emerging adults. We also analyzed the association between LAS7 scores and psychometric properties, which demonstrated the reliability of the LAS, but the correlations of LAS7 scores with various psychological scale scores were weak. The LAS is expected to be applied in future studies to address mental health among emerging adults, although further improvement and verification are needed.

## AUTHOR CONTRIBUTIONS

**Rumiko Kinkawa**: Conceptualization; data curation; validation; formal analysis; investigation; methodology; project administration; visualization; writing—original draft; writing—review and editing. **Miki Ono**: Conceptualization; data curation; validation; formal analysis; investigation; methodology; project administration; visualization; writing—original draft; writing—review and editing. **Satoshi Horiuchi**: Data curation; formal analysis; methodology; writing—original draft; writing—review and editing. **Masayuki Kikkawa**: Data curation; writing—original draft; writing—review and editing. **Shunichiro Ito**: Data curation; writing—original draft; writing—review and editing. **Akifumi Shimasaki**: Data curation; writing—original draft; writing—review and editing. **Yu Tamada**: Data curation; writing—original draft; writing—review and editing. **Jiro Masuya**: Data curation; writing—original draft; writing—review and editing. **Mina Honyashiki**: Data curation; writing—original draft; writing—review and editing. **Takeshi Inoue**: Data curation; writing—original draft; writing—review and editing.

## CONFLICT OF INTEREST STATEMENT

The authors have read the journal's policy and the authors of this manuscript have the following competing interests: Yu Tamada has received personal fees from Otsuka Pharmaceutical, Sumitomo Pharma, Eisai, MSD, and Meiji Seika Pharma. Jiro Masuya has received personal fees from Otsuka Pharmaceutical, Eli Lilly, Astellas, and Meiji Yasuda Mental Health Foundation, as well as grants from Pfizer. Takeshi Inoue has received personal fees from Mochida Pharmaceutical, Takeda Pharmaceutical, Janssen Pharmaceutical, Novartis Pharma, MSD, Yoshitomiyakuhin, Nipro, Kyowa Pharmaceutical Industry, Viatris, Lundbeck, Boehringer Ingelheim, Ono Pharmaceutical, and Meiji Seika Pharma; grants from Daiichi Sankyo and Tsumura; and grants and personal fees from Shionogi, Otsuka Pharmaceutical, Sumitomo Pharma, Mitsubishi Tanabe Pharma, and Eisai; and is a member of the advisory boards of Luye, Shionogi, GlaxoSmithKline, Viatris, and Otsuka Pharmaceutical. The remaining authors declare no conflicts of interest.

## ETHICS APPROVAL STATEMENT

The study protocol was approved by the Medical Ethics Review Committee of Tokyo Medical University (study approval number: T2022‐0053).

## PATIENT CONSENT STATEMENT

All participants were provided with a detailed study overview, including objectives, methodology, potential benefits, and risks. They were also informed that participation in the research was voluntary and that the data collected would be anonymized to ensure that the individuals could not be identified. Only the data of individuals who gave their consent to participate in this study were analyzed.

## CLINICAL TRIAL REGISTRATION

Not applicable.

## Supporting information

Supporting information.

Supporting information.

Supporting information.

## Data Availability

The data used in this study cannot be shared publicly because of Ethics Committee restriction. All relevant data are within the paper. Data are available from the Internal Review Board of the Department of Psychiatry, Tokyo Medical University, Japan (contact via email: seisinka@tokyo-med.ac.jp), for researchers who meet the criteria for access to confidential data.
