# Supplementary material for: Development of the Leisure Activity Scale for young adults: Reliability and validity
Source: PCN Rep. 2025 Mar 3;4(1):e70070. doi: 10.1002/pcn5.70070 (PMC11875056; doi:10.1002/pcn5.70070)
Supplement: Supplementary file 1 — Supporting information. [file PCN5-4-e70070-s002.docx]

**Supplementary Table 1 (English version of the Leisure Activity Scale)**

How much time do you usually spend doing the activities listed in categories (1) to (10) in the left column during your leisure time? Please circle the number of the frequency shown on the right that applies. In the left column, after the number, the category of the leisure time activity is indicated, followed by a description and specific examples in parentheses. If you do more than 1 activity within the same category, please answer about the activity that you most frequently engage in. If a leisure activity corresponds to more than 1 category, please put an answer for all the applicable categories (e.g., if you play sports with friends almost every day, answer 5 for both categories (1) and (5)).

| Activity category | Occasionally or not at all | 1 to 3 times a month | Once a week | Several days a week | Almost every day |
| --- | --- | --- | --- | --- | --- |
| **(1) Meeting and interacting type:** participating in a group activity/meeting with peers and friends  (dinner parties, hobby meetings, karaoke, chorus, ballroom dancing, events, board games/card games [shogi, mahjong, bingo, cards, karuta], online games, exchanging Line messages and emails, chatting, etc.) | 1 | 2 | 3 | 4 | 5 |
| **(2) Spiritual type:** aimed at obtaining spiritual and mental healing  (meditation, relaxation, aromatherapy, yoga, etc.) | 1 | 2 | 3 | 4 | 5 |
| **(3) Tour type:** to see scenery and products  (travel, sightseeing, city walking, shopping, driving, etc.) | 1 | 2 | 3 | 4 | 5 |
| **(4) Thinking/Learning type:** aimed at thinking and exploring  (puzzles, brain training, crossword puzzles, culture schools, workshops, lectures, lessons, learning languages) | 1 | 2 | 3 | 4 | 5 |
| **(5) Physical activities type:** aimed at physical exercise  (gymnastics, sports, gym workouts, competitions, outdoor activities, walking) | 1 | 2 | 3 | 4 | 5 |
| **(6) Cultural activities-Creative type:** those that aim to enjoy making things, such as artwork  (DIY, handicrafts, painting, making videos, playing music, writing, cooking/pastry making for fun) | 1 | 2 | 3 | 4 | 5 |
| **(7) Cultural activities-Appreciation type:** viewing and listening to artistic work regardless of location  (movies, music, paintings, going to the theater, etc.) | 1 | 2 | 3 | 4 | 5 |
| **(8) Social activities type:** engaging and participating in social activities  (volunteering, activities at temples and churches, playing with children, etc.) | 1 | 2 | 3 | 4 | 5 |
| **(9) Feeling nature type:** aimed at interacting with nature  (hiking, mountain climbing, horticulture, gardening, camping, fishing, spending time with pets, etc.) | 1 | 2 | 3 | 4 | 5 |
| **(10) Information gathering type:** activities to gather information through the media  (reading newspapers, watching TV, listening to the radio, watching recorded programs, reading books, reading magazines and comics, browsing the Internet and social networking sites, watching videos on the internet) | 1 | 2 | 3 | 4 | 5 |
