# Supplementary material for: Development of the Leisure Activity Scale for young adults: Reliability and validity
Source: PCN Rep. 2025 Mar 3;4(1):e70070. doi: 10.1002/pcn5.70070 (PMC11875056; doi:10.1002/pcn5.70070)
Supplement: Supplementary file 2 — Supporting information. [file PCN5-4-e70070-s001.pdf]

Supplementary Table 2 (Japanese version of the Leisure Activity Scale)

あなたはふだん、余暇の時間では、①～⑩の活動をどのくらいしていますか。あてはまる番号に〇をつけてください。数字の次には余暇の活動の名称、;のあとには説明、括弧の中には具体例があります。なお、同じ番号内の活動を複数行っている場合は、頻度の多い方を回答して下さい。1 つの活動が 2 つ以上の番号に該当する場合は、該当するすべての番号に回答してください（例：友達とスポーツをほぼ毎日行っている場合は①と⑤を 5 と回答）。

	たまにしかしない、あるいはまったくしない	月に1～3回	週に 1 回	週 に 数日	ほぼ毎日
**①集い型、交流型**；グループに入って参加する ／ 仲間や友人と集まるもの（食事会、趣味の会、カラオケ、コーラス、社交ダンス、イベント、盤ゲーム・カードゲーム《将棋、麻雀、ビンゴ、トランプ、カルタ》、オンラインゲーム、Line やメールなどのやりとり、おしゃべりなど）	1	2	3	4	5
**②スピリチュアル型**；霊的、精神的な癒しを得ることを目的とするもの（瞑想、リラクゼーション、アロマ、ヨガなど）	1	2	3	4	5
**③見学型**；風景や商品を見ることを目的とするもの（旅行、観光、街歩き、ショッピング、ドライブなど）	1	2	3	4	5
**④思考・探求型**；考えたり、探求したりすることを目的とするもの（パズル、脳トレ、クロスワード、カルチャースクール、講習会、講演会、お稽古、語学）	1	2	3	4	5
**⑤身体活動型**；体を動かすことを目的とするもの（体操、スポーツ、ジム、競技、野外活動、散歩）	1	2	3	4	5
**⑥文化活動：創作型**；作品などのものづくりを楽しむことを目的とするもの（DIY、手芸、絵を描く、動画を作る、演奏する、作文、楽しみのための料理・菓子作り）	1	2	3	4	5
**⑦文化活動：鑑賞型**；場所を問わず芸術作品を鑑賞・視聴する （映画、音楽、絵画、観劇など）	1	2	3	4	5
**⑧社会活動型**；社会活動に従事・参加する（ボランティア活動、お寺や教会での活動、子供と遊ぶなど）	1	2	3	4	5
**⑨自然ふれあい型**；自然と触れあうことを目的とするもの（ハイキング、山登り、園芸、庭仕事、キャンプ、釣り、ペットとすごすなど）	1	2	3	4	5
**⑩情報収集型**；メディアを通じて情報収集するための活動（新聞を読む、テレビをみる、ラジオを聞く、録画した番組をみる、読書、雑誌や漫画を読む、インターネットや SNS を見る、インターネットで動画をみる）	1	2	3	4	5
