# Supplementary material for: Development of the Leisure Activity Scale for young adults: Reliability and validity
Source: PCN Rep. 2025 Mar 3;4(1):e70070. doi: 10.1002/pcn5.70070 (PMC11875056; doi:10.1002/pcn5.70070)
Supplement: Supplementary file 3 — Supporting information. [file PCN5-4-e70070-s003.docx]

**Supplementary Figure 1. Scree plot of the Leisure Activity Scale**
